# A Modular Multirotor Unmanned Aerial Vehicle Design Approach for Development of an Engineering Education Platform

**DOI:** 10.3390/s21082737

**Published:** 2021-04-13

**Authors:** Denis Kotarski, Petar Piljek, Marko Pranjić, Carlo Giorgio Grlj, Josip Kasać

**Affiliations:** 1 Department of Mechanical Engineering, Karlovac University of Applied Sciences, 47000 Karlovac, Croatia; marko.pranjic111@gmail.com (M.P.); carlo.g.grlj@gmail.com (C.G.G.); 2 Department of Technology, Faculty of Mechanical Engineering and Naval Architecture, University of Zagreb, 10000 Zagreb, Croatia; petar.piljek@fsb.hr; 3 Department of Robotics and Production System Automation, Faculty of Mechanical Engineering and Naval Architecture, University of Zagreb, 10000 Zagreb, Croatia; josip.kasac@fsb.hr

**Keywords:** engineering education, multirotor UAV, modular design, EMMR platform, testing environment

## Abstract

The development of multirotor unmanned aerial vehicles (UAVs) has enabled a vast number of applications. Since further market growth is expected in the future it is important that modern engineers be familiar with these types of mechatronic systems. In this paper, a comprehensive mathematical description of a multirotor UAV, with various configuration parameters, is given. A modular design approach for the development of an educational multirotor platform is proposed. Through the stages of computer-aided design and rapid prototyping an experimental modular multirotor (EMMR) platform is presented. Open-source control system and a novel EMMR enable students to create and test control algorithms for various multirotor configurations. The presented EMMR platform is suitable for students to expand their educational objectives in aerial robotics and control theory.

## 1. Introduction

The development of mechatronic systems has enabled many applications, especially in the field of mobile robotics and autonomous vehicles. Of particular interest are the mechatronic systems of unmanned aerial vehicles (UAVs) which have experienced tremendous progress with the development of micro-electromechanical systems (MEMS) sensors, microcontrollers, batteries, and propulsion components. We can distinguish several categories of UAVs. The most commonly used are aircraft with fixed wings, which can be comparable in size with unmanned combat aerial vehicles (UCAVs), and a multirotor type of UAVs that can have a couple of watts of power up to a few tens of kilowatts. Multirotor UAVs are characterized as nonlinear, inherently unstable, multivariable, and high energy consumption systems. This type of UAV can perform vertical take-off and landing (VTOL), remain stationary (hover), and fly at a moderate speed. Such abilities make multirotors suitable for a wide range of missions like surveillance [1], precise agriculture [2], search and rescue [3], and many others. Most common (conventional) multirotor configurations consist of an even number of rotors arranged symmetrically in one (or more) parallel planes, such as quadrotor [4], hexarotor [5], or octorotor [6]. Conventional configurations are underactuated and strongly coupled systems, therefore they cannot move arbitrarily in 3D space. With proper selection of the multirotor configuration geometric arrangement, it is possible to get a fully-actuated aircraft system with decoupled position and orientation. Such configurations represent omnidirectional aerial robots that can perform complex and precise movements.

The scope of educational robotics is the subject of numerous researches and a large number of programs with associated platforms have been implemented in the curriculum at different levels of education. Educational programs in robotics for children of primary school age, mostly contain basic principles, and such robots are programmed using designed drag & drop software. An overview of existing literature in robotics education using robotics kits for the primary school level is given in [7]. The most commonly used robotic platforms are ground mobile robots with a differential drive configuration for which there are numerous kits with system upgrade options. At the high school level of education, the goal of the education program is on teaching physics, sensors, signals, and data acquisition in robotics and control. The programming at that level is basically implemented through C or Python, and it depends on the control components and program tasks. Additionally, software infrastructure can be based on robot operating system (ROS) [8,9]. At the university level of education, the field of robotics and control theory is covered extensively in mechatronics engineering field of study through several courses. For example, from the navigation point of view, the mentioned ground mobile robots can be upgraded with vision-based systems in order to introduce advanced algorithms for image processing and artificial intelligence techniques [10]. Furthermore, from the aspect of control systems education, various types of inverse pendulums [11] and ball-on beam/plate platforms [12] are used as laboratory test benches. These platforms are commonly used as experimental tools for educating more advanced control algorithms.

Multirotor UAVs are nowadays used in a large number of applications, and many new applications are expected in the future, which makes them interesting as educational tools. Multirotor educational platforms integrate existing educational objectives applied to ground mobile robots, but since they operate in three-dimensional (3D) space they are more demanding to design and control. Such aircraft are suitable to facilitate engineering education and research in the field of robotics and automation. Trends show that more and more multirotor platforms are being considered for the high school education level, such as a robotic platform for promoting programming and robotics skills [13]. Educational multirotor platforms for primary school level are also available on the market [14,15], with appropriate software that enables a quick start learning. Through the program, children learn block-based programming, the main principles of aerodynamics, and also use their critical thinking skills. Hardware-in-the-loop (HIL) platforms represent an effective approach in the design of UAVs since they are capable of running simulations and experiments [16]. Such platforms enable students and engineers to test and tune the control algorithms.

Several open-source platforms are used for research and engineering education, such as Parrot ArDrone quadrotor [17], or Crazyflie 2.0 nano quadrotor [18]. For education, other Parrot aircraft [19] are also eligible, and several other platforms such as IRIS quadrotor [16]. There are various papers that deal with the identification of propulsion parameters using test bench systems [20,21]. Furthermore, several approaches are investigated for the purpose of multirotor performance testing with experimental test rigs with three degrees of freedom (DOF) [22,23]. For the education purpose, experimental bench designs that can be made easily and cheaply are preferred, such as the setup presented in [24], and that can be easily upgraded with load cell sensors as presented in research [25]. Today’s technologies enable the development of experimental platforms like the quadrotor presented in [26]. The quadrotor is the most common multirotor UAV configuration and is often used as a platform for testing various control techniques, like proportional–integral–derivative (PID) control algorithms and linear–quadratic regulator (LQR) presented in [27], or more advanced techniques such as backstepping and sliding mode control (SMC) presented in [28]. Although mentioned educational platforms can be expanded with additional elements and modules, their propulsion system parameters are often fixed or very limited in terms of hardware components and aircraft configuration parameters. It is important to mention that a quadrotor can no longer achieve equilibrium in the event of a rotor failure, only perform a landing task. Using conventional configurations with more than four rotors, fault-tolerant control is possible, as proposed for the hexarotor configuration in [29]. Conventional multirotor configurations with the modular design that allows a change in configuration parameters are shown through work [30]. Although the proposed design is useful, the paper does not present experimental testing of the concept. Bearing in mind the possible different applications of multirotor UAVs, unconventional multirotor configurations have been considered and investigated [31,32], as well as fully-actuated multirotor configurations [33,34].

In this paper, a modular multirotor UAV design approach is presented for the development of an engineering (university level) education platform. A modular educational platform with complete open-source software is suitable for students to expand their educational objectives in robotics-mechatronics system design and control solutions. To enable simulations of system behavior and testing of different control algorithms a comprehensive mathematical model of a multirotor UAV for various configuration parameters is presented. Furthermore, the design steps of an experimental modular multirotor (EMMR) are given based on system characterization and the development process is presented through the phases of construction and manufacturing. The proposed modularized platform, from the aspect of system dynamics and control, combines the properties of commercial and experimental aircraft in one embodiment. Given the ability to change propulsion module parameters, it can potentially expand educational objectives to the area of omnidirectional aerial robots and the area of fault-tolerant control. Autonomous operation can also be achieved by adding additional sensors to sense the environment (lidar, ultrasonic, infrared, vision). Preliminary tests of EMMR in combination with test bench are given to perform mechanical load testing and to show some capabilities and usefulness for teaching and research purposes.

The paper is organized as follows: Section 2 present a mathematical model of a multirotor UAV system. The design concept of modular multirotor UAV is proposed in Section 3. The development and manufacturing of EMMR are presented in Section 4. In Section 5, the educational application and experiments are shown. Finally, the discussion and conclusions follow at the end.

## 2. Multirotor UAV Mathematical Description

Multirotors can be mathematically represented as rigid bodies with six DOF since they exist in 3D space. The only moving parts are the motor rotors on whose axes the fixed-pitch propellers are mounted, so it follows that the dynamics of this type of UAV directly depend on the rotor’s angular velocities. The symmetry of the aircraft body and the stiffness of the propeller were assumed. In this section, a comprehensive mathematical model of multirotor UAV is given. Multirotor UAV configuration is defined by the geometric arrangement of the rotors and by their components. The rotor represents an electric propulsion unit (EPU) consisting of an electronic speed controller (ESC) and a brushless DC (BLDC) motor to whose rotor a fixed-pitch propeller is attached.

**Figure 1:**
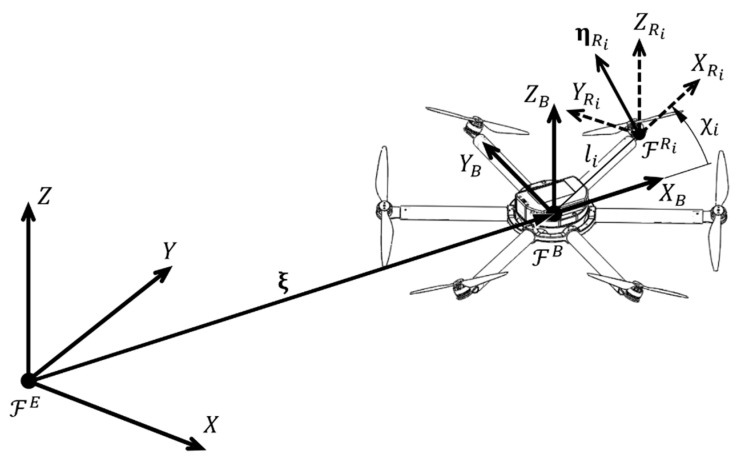
Multirotor reference coordinate systems.

From the aspect of the aircraft motion, the multirotor state is, among other quantities, defined by position and orientation in 3D space. In order to describe the aircraft motion, typical right-handed Cartesian coordinate systems are used, as shown in Figure 1. In a base station observed as a stationary point on the earth’s surface, an inertial coordinate system (Earth frame, ${ℱ}^{E}$) is defined. It consists of a longitudinal axis (X), a lateral axis (Y), and a vertical axis (Z) whose positive direction is defined upwards from the ground level. In terms of flight planning (waypoints or trajectory), ${ℱ}^{E}$ is a reference frame. The following is the aircraft coordinate system (body frame, ${ℱ}^{B}$) which is fixed to the moving multirotor UAV body. It is assumed that the ${ℱ}^{B}$ origin coincides with the center of gravity (COG), and that the axes ($X_{B}$, $Y_{B}$ and $Z_{B}$) coincide with the multirotor main axes of inertia. The equations of motion are described in the ${ℱ}^{B}$ since the inertia matrix is time-invariant. Propulsion (control) forces and torques are also defined with respect to the ${ℱ}^{B}$. Multirotor UAV position vector $\xi =\;{\left[\begin{array}{ccc} x & y & z \end{array}\right]}^{T}$ connects the origin of ${ℱ}^{E}$ with the origin of ${ℱ}^{B}$ as is shown in Figure 1. Multirotor UAV orientation (attitude) vector $\eta ={\left[\begin{array}{ccc} \phi & \theta & \psi \end{array}\right]}^{T}$ consist of three Euler angles. Roll angle ϕ is defined as rotation around the X axis, pitch angle θ is defined as rotation around the Y axis, while yaw angle ψ is defined as rotation around the Z axis. The multirotor UAV velocity vector $\nu ={\left[\begin{array}{cc} {v^{B}}^{T} & {\omega^{B}}^{T} \end{array}\right]}^{T}$, consisting of translational $v^{B}={\left[\begin{array}{ccc} u & v & w \end{array}\right]}^{T}$ and rotational $\omega^{B}={\left[\begin{array}{ccc} p & q & r \end{array}\right]}^{T}$ velocity vectors, is defined in ${ℱ}^{B}$. Finally, the coordinate system of the ith rotor (${ℱ}^{R_{i}}$) is defined, which allows a description of the allocation of rotors aerodynamic forces and moments to the propulsion (control) vector.

A rigid body is an idealized body of invariant shape and volume in which the mutual position of the particles does not change. From these observations, it results that the movement of a rigid body can be described as the movement of a particle located in the body COG. Therefore, the motion of a multirotor can be described by the translation and rotation of ${ℱ}^{B}$ with respect to ${ℱ}^{E}$. The kinematics of a rigid body with six DOF is defined by the following expression:

(1) $$\dot{\varepsilon }=\Theta \nu$$

where the velocity vector $\dot{\varepsilon }={\left[\begin{array}{cc} {\dot{\xi }}^{T} & {\dot{\eta }}^{T} \end{array}\right]}^{T}$ is defined in ${ℱ}^{E}$. It is obtained using the overall mapping matrix:

(2) $$\Theta =\left[\begin{array}{cc} R & 0_{3\times 3}\\ 0_{3\times 3} & \Omega_{B} \end{array}\right]$$

where R is the rotation matrix, $0_{3\times 3}$ is the zero matrix, and $\Omega_{B}$ is the transformation matrix. Rotation matrix maps the translational velocities from ${ℱ}^{B}$ to ${ℱ}^{E}$ which is described, according to Euler’s orientation theorem, by three consecutive rotations (yaw ψ—pitch θ—roll ϕ). The orthogonal rotation matrix is given with the following expression:

(3) $$R=\left[\begin{array}{ccc} c_{\psi }c_{\theta } & c_{\psi }s_{\theta }s_{\phi }-s_{\psi }c_{\phi } & c_{\psi }s_{\theta }c_{\phi }+s_{\psi }s_{\phi }\\ s_{\psi }c_{\theta } & s_{\psi }s_{\theta }s_{\phi }+c_{\psi }c_{\phi } & s_{\psi }s_{\theta }c_{\phi }-c_{\psi }s_{\phi }\\ -s_{\theta } & c_{\theta }s_{\phi } & c_{\theta }c_{\phi } \end{array}\right]$$

where $c_{i}=\cos\left(i\right),{\;s}_{j}=\sin\left(j\right)$. Transformation matrix maps rotational velocities from ${ℱ}^{B}$ to ${ℱ}^{E}$, which is described by resolving Euler angle rates:

(4) $$\Omega_{B}=\frac{1}{c_{\theta }}\left[\begin{array}{ccc} c_{\theta } & s_{\phi }s_{\theta } & c_{\phi }s_{\theta }\\ 0 & c_{\phi }c_{\theta } & -s_{\phi }c_{\theta }\\ 0 & s_{\phi } & c_{\phi } \end{array}\right]$$

### 2.1. Multirotor UAV Dynamic Model

The dynamic model of the multirotor UAV is described by the aircraft equations of motion with respect to ${ℱ}^{B}$ and by the forces and moments acting on the aircraft system. Equations of motion are represented with a system of six second-order differential equations which are derived using the Newton-Euler approach. The equations of motion are given in matrix form:

(5) $$M_{B}\dot{\nu }+C_{B}\left(\nu \right)\nu =\Lambda$$

where $M_{B}$ is a rigid body inertia matrix, $C_{B}\left(\nu \right)$ Coriolis and centripetal matrix, and Λ vector of forces and moments acting on a rigid body.

Rigid body inertia matrix comprises members that resist body motion. It is time-invariant (${\dot{M}}_{B}\;=\;0$) and its parameterization is unique. It is given by the following expression:

(6) $$M_{B}=\left[\begin{array}{cc} mI_{3} & 0_{3\times 3}\\ 0_{3\times 3} & I \end{array}\right]$$

where m is the multirotor UAV mass, $I_{3}$ identity matrix, and $I=\mathrm{diag}\left\{\begin{array}{ccc} I_{\mathrm{xx}}, & I_{\mathrm{yy}}, & I_{\mathrm{zz}} \end{array}\right\}$ body’s inertia matrix since it is assumed that the ${ℱ}^{B}$ origin coincides with the COG, and that the axes coincide with the multirotor main axes of inertia.

Coriolis and centripetal matrix describes the inertial forces with respect to the rotating ${ℱ}^{B}$. It is possible to parameterize it in different ways [35], where $C_{B}\left(\nu \right)=-C_{B}{}^{T}\left(\nu \right)$. It is given by the matrix representation of vector product a×b=S(a)b, where S is a skew-symmetric matrix:

(7) $$C_{B}\left(\nu \right)=\left[\begin{array}{cc} 0_{3\times 3} & -S\left(mv^{B}\right)\\ 0_{3\times 3} & -S(I\omega^{B}) \end{array}\right]$$

The multirotor dynamic is influenced by propulsion and external (environment) forces and moments. The model examines the impact of four influences that make up the overall vector of forces and moments while the other influences, in this case, are neglected, but can be subsequently considered and integrated into the model. Vector of forces and moments is given in vector form:

(8) $$\Lambda =g_{B}+o_{B}+d+u_{B}$$

where $g_{B}$ is the gravitational force vector which acts only on translational dynamics, $o_{B}$ the gyroscopic moment vector which acts only on rotational dynamics, d vector of disturbances, and $u_{B}$ propulsion (control) vector. The disturbance vector represents external disturbances, such as wind gusts, and unmodelled system dynamics. The overall dynamic model of the system is defined by the following matrix form expression:

(9) $$\dot{\nu }=M_{B}{}^{-1}\left(-C_{B}\left(\nu \right)\nu +g_{B}+o_{B}+d+u_{B}\right).$$

Through the propulsion vector, it is possible to directly influence the dynamics of the multirotor UAV system by changing the angular velocities of the rotors. Therefore it is called a control vector and consists of three forces and three moments $u_{B}={\left[\begin{array}{cc} f^{T} & \tau^{T} \end{array}\right]}^{T}={\left[\begin{array}{cccccc} f_{X} & f_{Y} & f_{Z} & \tau_{\phi } & \tau_{\theta } & \tau_{\psi } \end{array}\right]}^{T}$, one for each DOF. In this study, it is described by the following expression:

(10) $$u_{B}=\Gamma_{B}\Omega$$

where $\Gamma_{B}$ is the matrix of the control allocation scheme for aircraft configurations with various parameters (rotor position, cant and tilt angle), introduced in [36]. Propulsion control forces and moments are modeled as proportional to the square of the rotors angular velocities, $\Omega ={\left[\begin{array}{c} \omega_{1}{}^{2}\;\omega_{2}{}^{2}\ldots \;\omega_{N}{}^{2} \end{array}\right]}^{T}$.

### 2.2. Generalized Control Allocation Scheme

The control allocation scheme describes the mapping of the rotors’ angular velocities to the aircraft control vector since propellers mounted on rotors by their rotation create aerodynamic forces and moments required for the aircraft motion. Therefore, the propulsion configuration, respectively multirotor configuration, consisting of *N* rotors is described by a control allocation scheme. It is determined by the parameters of the rotors geometric arrangement and the power of each rotor. In practice, conventional configurations are used to perform various missions, and what they have in common is that they consist of an even number of symmetrically arranged rotors in one or more parallel planes. In order to cancel the reactive moment around $Z_{B}$, half of the rotors rotate in a clockwise (CW) direction, while the other half rotate counterclockwise (CCW). Additionally, there are configurations with the non-planar geometric arrangement, and of particular interest are the fully-actuated configurations, such as configuration with six rotors considered in [37]. The propulsion configuration geometric arrangement is defined by the position vector $\xi_{R_{i}}$ and orientation vector $\eta_{R_{i}}$ of each rotor. The position vector of the *i*th rotor is defined as:

(11) $$\xi_{R_{i}}=R_{R_{i}}{}^{T}\left(\chi_{i},\;Z_{B}\right)\left[\begin{array}{c} l_{i}\\ 0\\ 0 \end{array}\right]$$

where $\chi_{i}$ is the ith rotor position angle placed in the horizontal ($X_{B}Y_{B}$) aircraft plane, and $l_{i}$ is the *i*th rotor arm length, as shown in Figure 1. The orientation vector of the *i*th rotor is defined as:

(12) $$\eta_{R_{i}}=\;R_{R_{i}}{}^{T}\left(\chi_{i},Z_{B}\right)R_{R_{i}}{}^{T}\left(\beta_{i},\;Y_{R_{i}}\right)R_{R_{i}}{}^{T}\left(\gamma_{i},\;X_{R_{i}}\right)e_{3}$$

where $\beta_{i}$ is the *i*th rotor cant angle, $\gamma_{i}$ is the *i*th rotor tilt angle. Since the considered aerodynamic effects are represented in the rotor vertical axis, the expression uses a unit vector $e_{3}={\left[\begin{array}{ccc} 0 & 0 & 1 \end{array}\right]}^{T}$. The geometric arrangement parameters determine the allocation of rotors aerodynamic forces and moments on the control vector of the aircraft.

The control vector is the sum of the vectors of forces and moments of each rotor. Control forces and moments are modeled as proportional to the square of the rotor’s angular velocities, where rotors are represented with thrust force factor ($k_{f_{i}}$), and drag torque factor ($k_{\tau_{i}}$). The force vector of the *i*th rotor with respect to ${ℱ}^{B}$ is obtained by mapping the rotor thrust force using the orientation vector, and is defined by the expression:

(13) $$f_{i}=\left(k_{f_{i}}\eta_{R_{i}}\right)\omega_{i}{}^{2}$$

The moment vector of the *i*th rotor with respect to ${ℱ}^{B}$ consists of two components. The first component arises from the action of the thrust force, and is calculated by the vector product of the rotor position and orientation. The second component arises from the action of the drag torque and depends on the rotor orientation. The moment vector of the *i*th rotor derived by the matrix representation of the vector product is defined by the expression

(14) $$\tau_{i}=\left(k_{f_{i}}S\left(\xi_{R_{i}}\right)\eta_{R_{i}}+k_{\tau_{i}}\eta_{R_{i}}\right)\omega_{i}{}^{2}$$

The drag torque factor ($k_{\tau_{i}}$) sign depends on the rotor direction, where CW rotors have a positive sign, while CCW rotors have a negative sign.

Control allocation matrix ($\Gamma_{B}\in {\mathbb{R}}^{6\times N}$) for multirotor UAV configuration is defined with the following expression:

(15) $$\Gamma_{B}=\left[\begin{array}{ccc} k_{f_{1}}\eta_{R_{1}} & \ldots & k_{f_{N}}\eta_{R_{N}}\\ k_{f_{1}}S\left(\xi_{R_{1}}\right)\eta_{R_{1}}+k_{\tau_{1}}\eta_{R_{1}} & \ldots & k_{f_{N}}S\left(\eta_{R_{N}}\right)\eta_{R_{N}}+k_{\tau_{N}}\eta_{R_{N}} \end{array}\right]$$

where *N* is a number of rotors. Since the multirotor is represented as a rigid body with 6 DOFs, it follows that the allocation matrix has 6 rows, one for each DOF. The number of rotors defines the number of matrix columns that represent the parameters of each rotor. The control allocation matrix rank determines the number of controlled DOF of the multirotor system, which directly affects the control design and flight planning. When the rank of the matrix is not in full order, the configuration is underactuated, which is characteristic of conventional aircraft. Configurations with the full rank of the allocation matrix are fully-actuated, so they can accelerate in any direction in space.

In terms of control system implementation on a real aircraft with various configuration parameters, it is necessary to calculate the inverse matrix, commonly referred to as a motor mixer. The outputs of the control algorithm represent a control vector, therefore it is necessary to allocate those outputs to the angular velocity of each rotor. Since aircraft with less or more than six rotors do not have a square allocation matrix, angular velocities of the rotors are calculated using pseudoinverses of the allocation matrix as given with the following expression:

(16) $$\Omega =\Gamma_{B}{}^{T}{\left(\Gamma_{B}\Gamma_{B}{}^{T}\right)}^{-1}u_{B}$$

The presented multirotor UAV mathematical model can be easily implemented in a software package and can be used to simulate the aircraft behavior with various configuration parameters. The mathematical model can be used in education as a tool for the analysis of aircraft parameters, selection of real system components, evaluation and optimization of propulsion geometric arrangement parameters. Furthermore, the inverse of the control allocation scheme allows the control implementation of modular aircraft.

## 3. Modular Multirotor UAV Design Considerations

To ensure overall flight performance, it is necessary to determine the required thrust-to-weight ratio (TWR). As a rule, multirotor UAVs are designed with approximately twice the thrust of the weight itself, where a more precise ratio is recommended by propulsion manufacturers. It can be concluded that the mass of the aircraft is a very important parameter in the design of such a system. For simpler analysis, design, and development of this type of UAV, the aircraft system is divided into four key subsystems regardless of the configuration or purpose of the aircraft. The total mass of the aircraft system is then equal to the sum of the masses of all its subsystems:

(17) $$m=m_{AV}+m_{PS}+m_{ES}+m_{PL}$$

where $m_{AV}$ is the mass of the control subsystem (avionics), $m_{PS}$ the propulsion subsystem mass, $m_{ES}$ the energy subsystem mass, and $m_{PL}$ is the mass of the payload equipment and cargo. The design of a modular aircraft involves trade-offs between key subsystems, i.e., modules so it is necessary to analyze all vehicle subsystems that affect each other concerning the required flight tasks. The subsystems are mechanically connected to each other by frame construction parts which must be light, solid and rigid, and easy to connect. Parts of the frame construction together with the components form sub-assemblies of each subsystem which together form the assembly of the aircraft.

**Figure 2:**
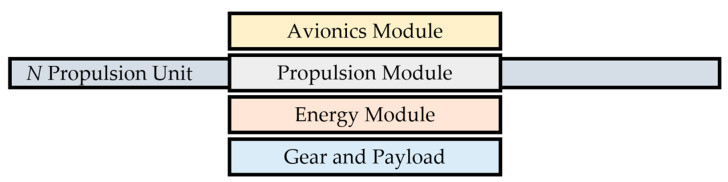
Modular multirotor topology.

With the aim of testing multiple aircraft configurations, the idea of a modular multirotor UAV was born. There are several papers on this topic, like multipurpose modular drone produced via the additive manufacturing process [30], or modular multirotor system for a flight of rigid objects presented in [38]. Guidelines for the development of the proposed modular concept are given by mentioned four key subsystems. In the phase of design and development, the subsystems are transformed into four modules. The modular concept should allow for the multi-purpose character of the aircraft, depending on the needs within the area of application. The constructed frame parts should allow easy assembly/disassembly of the aircraft system. Since the flight performance is determined by the propulsion and energy module, the greatest emphasis will be on the design solutions for them. In the proposed concept, the four modules form the central part of the aircraft, where the propulsion module comprises propulsion units connected by arms to the central assembly. Figure 2 shows the possible and further considered topology of a modular multirotor UAV. Conveniently, the propulsion and energy modules are adjacent so that the distribution of energy from the batteries to the propulsion units can be efficiently carried out, and to achieve a favorable mass distribution of the system.

The advantage of a modular approach to aircraft design is also its scalability. The same approach can be applied to small aircraft with a power of several hundred watts up to large aircraft with a power of several tens of kilowatts. For educational purposes, the development of a small modular aircraft with a maximum power of 800 W is further considered and presented.

### 3.1. Multirotor UAV Propulsion Module

**Figure 3:**
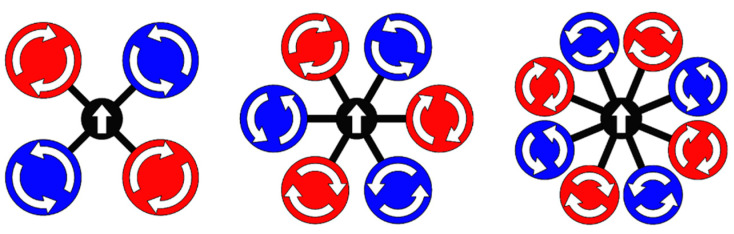
Conventional multirotor configurations.

From the aspect of system dynamics, the propulsion module should provide the necessary forces and moments for the aircraft motion in 3D space. Therefore, the multirotor UAV flight performance depends on the geometric arrangement and power of the rotors. The basic geometric parameters of conventional configurations are the number of rotors (*N*), and the rotor propeller diameter (*d*). The most common variants of conventional configurations are shown in Figure 3. In this paper, the EMMR, whose size is defined by a diameter of *D* = 500 mm is designed and developed.

The electric motor type of propulsion module is considered due to the ability of precise and fast control. The high reliability of electric propulsion reduces the possibility of multirotor UAV crash in case of propulsion failure. Propulsion configuration and its components need to be properly selected to ensure the required aircraft performance, which depends on the mission profile. The propulsion module consists of *N* EPUs located at the ends of the rotor arms.

**Figure 4:**
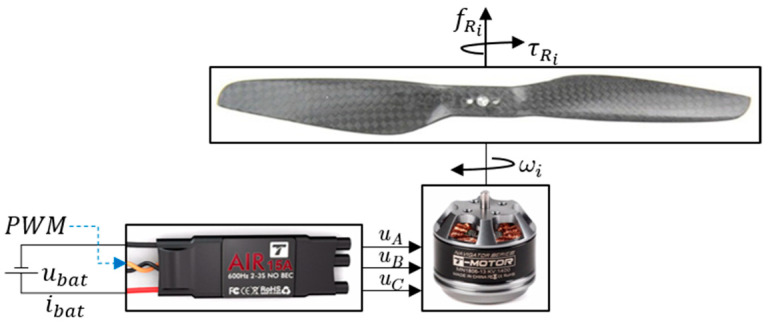
EPU components.

**Figure 5:**
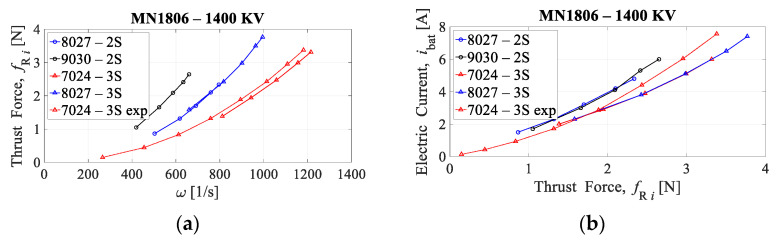
EPU characteristics: (**a**) thrust force as a function of rotor angular velocity; (**b**) electric current as a function of thrust force.

The EPU, as shown in Figure 4, consists of an ESC and a mechanical assembly of the BLDC motor on whose rotor a fixed-pitch propeller is mounted. The thrust force ($f_{R_{i}}$) and the drag torque ($\tau_{R_{i}}$) of ith rotor depend on the angular velocity of that same rotor ($\omega_{i}$). For the considered EMMR, the EPUs (rotors) consist of Tiger MN1806 BLDC motor (T-MOTOR, Nanchang, China) that can be combined with a wide range of propeller diameters (from 5″ to 9″), where diameters from 7″ to 9″ are considered for the further design of EMMR. Additionally, the MN1806 motor design allows propeller mounting in pusher and puller variants, so configurations with overlapping propeller surfaces are possible [39]. Based on the manufacturer’s specification for considered EPU, the characterization was performed, which is presented in previous work [40]. In this paper, four setups of EPUs were considered, and based on propulsion characterization, two EPU setups are chosen which will be paired with the energy subsystem i.e., batteries with three (3S) cells. Figure 5 shows thrust force with respect to rotor angular velocity, and electric current with respect to thrust force. The first two numbers in the graph legend indicate the propeller diameter, while the next two indicate the propeller pitch, both in inches. The characteristics are based on the propulsion component manufacturer’s specification [41]. For more accurate characterization, it is desirable to perform experimental measurements as shown in the graphs for a setup 7024-3S exp.

### 3.2. Multirotor UAV Energy Module

The energy module should provide sufficient energy to the propulsion module in order to enable the required endurance. The energy requirements of the propulsion module must be taken into account since the multirotor type of UAV is characterized by high energy consumption. The mass of the overall multirotor UAV system directly affects the selection of the propulsion and energy modules, therefore these two modules are interdependent. When choosing components for each module it is necessary to maintain a balance in order to satisfy the required performance with the existing constraints. EPUs based on BLDC motors are combined with an energy module consisting of lithium-polymer (LiPo) batteries. Additionally, the energy module consists of components for distributing energy to the control module.

LiPo batteries are rechargeable high energy density batteries that use lithium ions to transfer charge between electrodes. Such type of batteries consists of one or more electrochemical cells where the nominal voltage of each battery cell is 3.7 V, and the fully charged cell is 4.2 V. They are characterized by a high discharge rate in order to provide a continuous energy stream to the propulsion module. The main parameters that define LiPo batteries are capacity (in mAh), discharge rate (C), and the operating voltage i.e., the number of cells (S). When choosing batteries, the crucial is the relationship between battery mass (weight) and capacity. For further analysis and implementation, Table 1 shows the considered batteries and their parameters.

### 3.3. Mass Distribution of the Aircraft System

**Figure 6:**
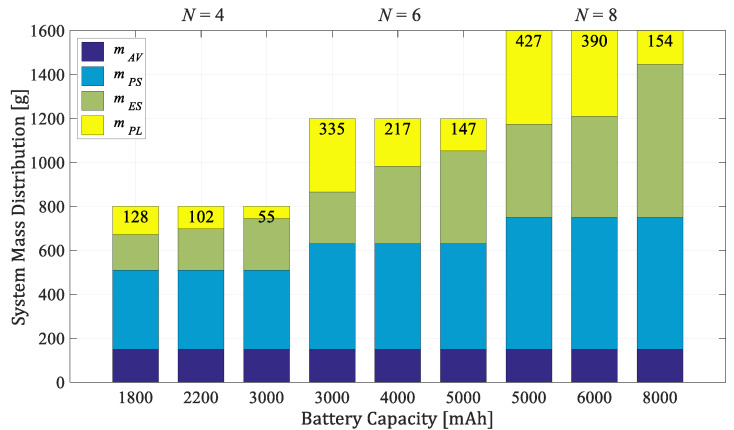
Mass distribution of the aircraft series with 7024 propellers and 3S batteries (TWR = 1.7).

**Figure 7:**
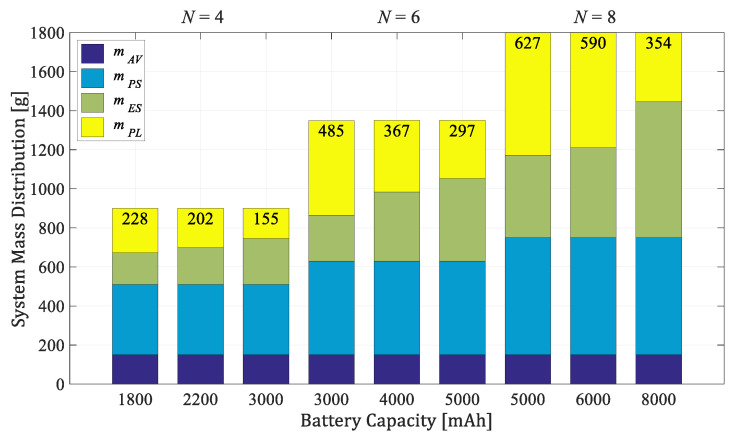
Mass distribution of the aircraft series with 8027 propellers and 3S batteries (TWR = 1.7).

The relationship between the propulsion module total thrust and the weight of the overall system, defined as TWR, is determined according to the manufacturer’s recommendations, and for considered EPU, TWR = 1.7 [41]. Figure 6 and Figure 7 show the mass distributions of payload mass ($m_{PL}$), energy subsystem mass ($m_{ES}$), propulsion subsystem mass ($m_{PS}$), and control subsystem (avionics) mass ($m_{AV}$) for two considered series of conventional configurations consisting of four, six, and eight rotors. The energy module consists of one or more 3-cell batteries. The first series (Figure 6) is based on an EPU setup with a 7024 propeller, while the second (Figure 7) contains an 8027 propeller. As seen from the graphs, the largest mass has the propulsion subsystem, where the propulsion unit is approximately 60 g, and the energy subsystem which primarily depends on the battery’s mass (Table 1). The proposed concept allows for a wider range of potential applications as parameter changes affect payload capacity, agility, and endurance.

## 4. Development and Rapid Prototyping of EMMR

**Figure 8:**
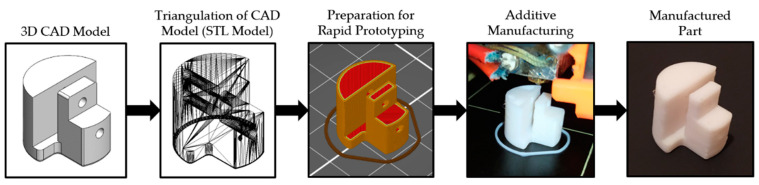
Framework for integrated design and rapid prototyping.

In the design phase of the experimental aircraft, the Solidworks software package (Dassault Systèmes SE, Vélizy-Villacoublay, France) was used for the 3D modeling of parts. In the development phase, rapid prototyping technologies were used for manufacturing parts in order to shorten the production process and reduce costs. The choice of construction materials depends on the purpose of each part of the system. Generally, the main requirements are high strength and rigidity and low specific weight. A 3-axis computer-controlled milling machine was used to make the parts by the cutting process, mainly from a plate of composite materials. Additive technologies, as an essential element in the teaching of robotics [43], were employed to make plastic parts of polymer, using the selective laser sintering (SLS), and the fused deposition modeling (FDM) 3D printing process. To create parts by SLS process it was utilized the Formiga P110 high-performance additive manufacturing system (EOS GmbH, Krailling, Germany) and parts were made of EOS PA2200 material. On the other hand, the MK2S desktop printer (Prusa, Prague, Czech Republic) was used to make the parts by the FDM process, mainly of PLA material. Figure 8 shows a framework in which the design process and rapid prototyping are integrated. The framework shows the phases of the part manufactured by the FDM process, that goes into the carbon tube of the rotor arm assembly. The result of the design phase is an aircraft assembly consisting of four modules. During the development phase, parts based on the CAD models were manufactured in order to assemble experimental aircraft.

### 4.1. Development and Manufacturing of EMMR Propulsion Module

The development of the propulsion module is the first and most important step in the development of the entire aircraft. The main goal is to design a propulsion module that allows the assembling of as many aircraft configurations as possible. The presented modular propulsion module consists of a central assembly to which other aircraft modules and rotor arms are connected. Designed central assembly of the propulsion module has features such as the mechanical and electrical connection of other modules and rotor arms. Commercially available connectors (XT30 type and XT60 type), are considered for energy distribution, while male/female headers are considered for signal distribution, both integrated into the aircraft frame parts and elements. The mechanical connection of the frame parts is realized using bolted joints.

**Figure 9:**
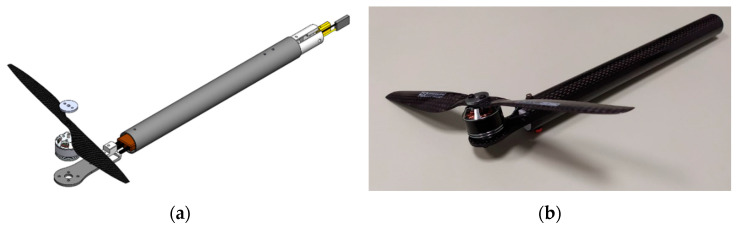
Rotor arm: (**a**) CAD assembly; (**b**) prototype assembly.

First, the development of the rotor arm for the considered EPU based on the MN1806 BLDC motor is shown. It is necessary to design and manufacture parts on which the EPU components are mounted and which enable the rotor arm connection to the central assembly of the propulsion module. The MN1806 motor is mounted on a carbon fiber plate adapter whose geometry depends on the motor dimensions. The motor adapter plate is coupled via a plastic adapter to the prefabricated carbon fiber tube inside which the ESC is located. The other side of the tube consists of a plastic adapter into which the male XT30 connector and the ESC signal connector are incorporated. Figure 9 shows a 3D model of the rotor arm assembly and the rotor arm prototype.

**Figure 10:**
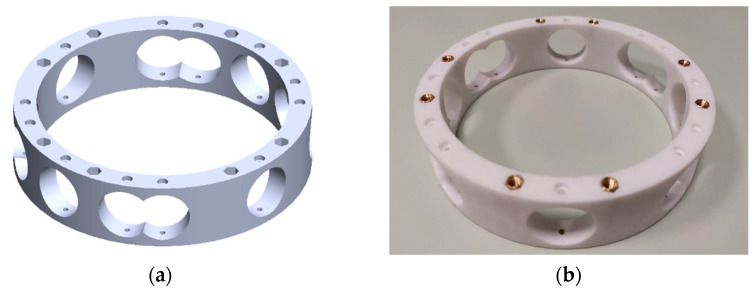
Propulsion center inner ring: (**a**) CAD model; (**b**) manufactured assembly.

The rotor arms are mounted on the central assembly of the propulsion module which allows the distribution of energy (power) and signal. Power is distributed via a high-power circuit that connects the batteries to the EPUs via the incorporated XT30 and XT60 connectors with an integrated power sensor that supplies power and signals to the control module. The distribution of signals from the control module to the rotor arms and the low-power circuit that connects peripheral sensors, signal lights, and other equipment, is achieved using the manufactured elements. A prototype of the propulsion module central assembly is considered, which enables the connection of an even number of rotors symmetrically arranged in the horizontal ($X_{B}Y_{B}$) aircraft plane. The basic part of the central assembly is the inner ring (Figure 10) whose geometry defines the configurations of the aircraft, and on which other aircraft modules are mounted. This enables the utilization of six conventional propulsion configurations (quadrotor +, quadrotor X, hexarotor +, hexarotor X, octorotor +, and octorotor X). In addition, with the two considered EPU setups, the total number of configurations is 12.

**Figure 11:**
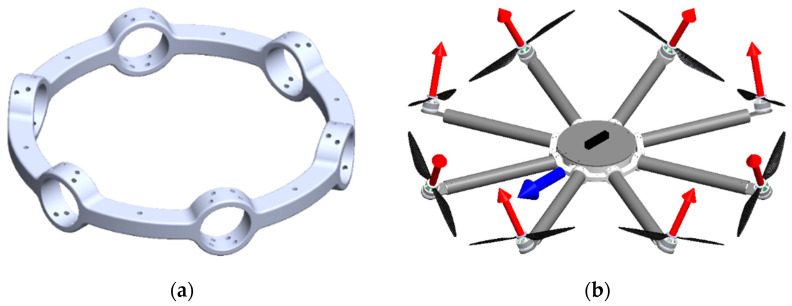
Propulsion module outer ring: (**a**) Part model of the propulsion assembly for fully-actuated configuration with six rotors; (**b**) Assembly model of the propulsion subsystem for fully-actuated configuration with eight rotors.

In this research of particular interest are fully-actuated multirotor configurations representing omnidirectional aerial robots. Such aircraft can perform complex and precise movements which is of crucial importance in missions involving interaction and manipulation. For the purpose of testing fully-actuated multirotor configurations, a solution with an additional propulsion module outer ring is considered. The outer ring feature allows passive adjustment of the rotor tilt angle ($\gamma_{i}$), thereby determining the allocation of aerodynamic forces and torques to the aircraft control vector. Outer rings for configurations with six (Figure 11a) and eight (Figure 11b) rotors, with four possible tilt angles (15°, 20°, 25°, and 30°) were designed and manufactured.

### 4.2. EMMR Assembly

The EMMR aircraft assembly consists of the presented propulsion module, the control module (avionics), the energy module, and the equipment module. In the case of an aircraft intended for experimental testing, the equipment module actually represents the landing gear. In a special case for testing the aircraft attitude, it is developed gimbal joint assembly.

**Figure 12:**
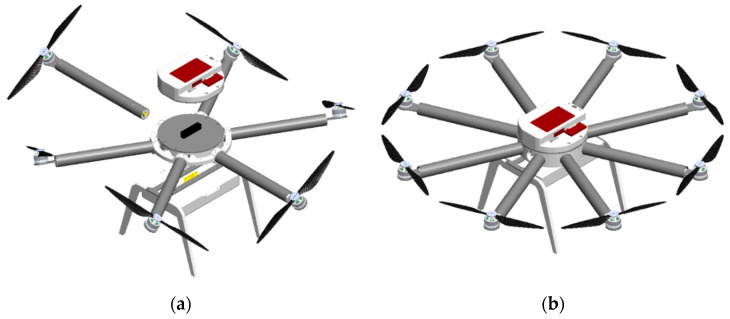
Multirotor configurations 3D models: (**a**) non-flat hexarotor X (NFX6); (**b**) flat octorotor X (FX8).

The control module is based on an open-source PX4 (Dronecode Foundation, San Francisco, CA, USA) flight controller (FC). For the two types of PX4 FC, discussed in the next section, parts of the control module housing have been developed. Peripheral components of the FC and remote control elements are incorporated inside the housing. The control module is connected to the top of the propulsion module as shown in Figure 12a. The energy module, which in this case consists of two LiPo batteries, is connected to the propulsion module from the bottom. Figure 12b shows a proposed 3D model of a conventional eight-rotor configuration, the so-called flat octorotor (FX8). Flat-layout configurations, regardless of the number of rotors, are classified as underactuated multirotor configurations. Therefore, such systems are strongly coupled and thus more demanding to control. Additionally, the modular aircraft design allows the assembling of fully-actuated aircraft configurations such as non-flat hexarotor (NFX6), whose embodiment is shown in Figure 12a.

For the educational platform as presented in the paper, all parts, except the prefabricated carbon tubes, can be made by additive manufacturing. Moreover, due to the size and power of the educational aircraft, parts can be made by using FDM technology that is available in almost all technical universities and institutes, so the proposed approach can be applied in-house. Also, in case of a crash only damaged parts or components need to be replaced, and since multirotor parts are mostly made with ubiquitous 3D printing technologies, replacement parts are easily produced on demand. Therefore, the proposed novel EMMR represents a versatile and reproducible solution for education in the field of aerial robotics and automation, given that configurations with different parameters can be assembled in one embodiment.

## 5. Educational Applications

Since multirotor UAVs exist in 3D space, compared to ground-based mobile robots, they are more demanding from the aspect of overall system design. Considering the control allocation scheme, it is possible to analyze the influence of propulsion system parameters on aircraft performance for different configurations. This further enables the selection of suitable components and optimization of parameters regarding the requirements in individual missions and constraints in the design itself. Since fully-actuated configurations can be assembled, this capability extends educational applications to the field of omnidirectional aerial robots that, unlike conventional configurations, can move in arbitrary directions in 3D space. Furthermore, the EMMR design allows easy integration of additional system features, such as distance sensors or vision systems, which further expands the potential educational applications of the platform. Since the EMMR platform is planned to be used as an educational tool in the new course on UAV mechatronics, Table 2 provides an overview of the possibilities where the educational objectives are given as control objectives. Modular design allows easy implementation with test rigs and testing of independent control with one, two, three, four, and six DOF. From the aspect of fault-tolerant control, due to the versatility of the propulsion module, EMMR is a powerful educational tool for education through testing of various cases (various number and geometric arrangement of rotors, various number of rotor failure, and various position of rotor failure).

Control system design, which includes a field of control theory, signal processing, and other related fields, represents a considerable area of educational application. Multirotor type of UAV is generally a very interesting platform for evaluating existing and testing new control approaches which are part of many curriculums in engineering education. Furthermore, the control methods taught at the University of Zagreb through existing courses are considered in a new course on UAV mechatronics. Therefore, PID control, feedback linearization control (FLC), and robust control methods such as the SMC and active disturbance rejection control (ADRC) are planned to be taught through a new course. In real systems such as multirotor UAV, the field of control theory is closely related to the field of signal processing. The data from the sensor is noisy and needs to be processed. As with the implementation of control algorithms, the same applies to the implementation of various signal filters and state estimators. It follows that a multirotor aircraft can be used for educational purposes as a benchmark for different types of filters. For instance, a simple complementary filter or built-in extended Kalman filter (EKF). Furthermore, existing state and disturbance estimators, such as linear extended state observer (ESO) or novel state estimators, can be taught.

Below is a proposal for the exercises that are planned to be incorporated into the syllabus for the new course on UAV mechatronics at the study of mechatronics at the University of Zagreb.

- Attitude control exercise—include synthesis of cascade PID controller, implementation on a real system, tuning procedure of controller parameters, experimental tests of various conventional configurations in combination with a test bench.
- Position control exercise—include implementation of remote control and GPS modules, experimental flights with various underactuated configurations, performance analysis.
- Trajectory tracking control exercise—include synthesis of FLC, implementation for underactuated and fully-actuated configurations, state estimation, flight planning, experimental flights with various underactuated and fully-actuated configurations.
- Disturbance rejection control exercise—include synthesis of control algorithm based on SMC, implementation, comparison with PID and FLC, experimental tests of various configurations in combination with a test bench and external disturbance.
- Fault-tolerant control exercise—include active disturbance rejection control design based on algebraic ESO [44] for underactuated and fully-actuated configurations, implementation, experimental tests of various cases of a propulsion module failure.

In potential applications of the aircraft where interaction with the environment, external disturbances, or even failure of the propulsion subsystem is possible, it is extremely important from a safety aspect that the system consists of safety mechanisms. In addition to mechanical mechanisms such as parachute, there are also control mechanisms in the form of fault-tolerant control algorithms. Therefore the area of fault-tolerant control is becoming increasingly interesting and important for future engineers, given that aircraft safety is a necessary prerequisite for utilization. The versatility of the proposed EMMR platform allows teaching fault-tolerant control in more detail. An example of an experiment included in the fault-tolerant control exercise is testing of a fully-actuated octorotor configuration in the case of performing complex movements (e.g., following a 6 DOF trajectory) with a failure for one or two rotors. Furthermore, in contrast to similar platforms, the proposed EMMR is a fully functional multirotor UAV with the capability of problem-solving real-world scenarios outside the classroom, which raises the attention of students and improves their learning capabilities.

In the experimental phase, the safest verification of the system is by integrating the aircraft with the test-bench (gimbal joint), which is very convenient given that the aircraft is intended to be used as an educational tool. This allows for a phase of laboratory testing and adjustment of the control system and prevents aircraft from crashing due to software and hardware bugs. The proposed EMMR platform is currently used in the research. The same concept of a modular aircraft has been used for the construction of several other aircraft, also used in research. The proposed aircraft has proven to be very practical as it allows relatively fast disassembly and assembly of modules so no unnecessary time is wasted and all commonly used underactuated and fully-actuated configurations can be assembled. The next step necessary before the development of education is detailed and extensive testing on the basis of which the course implementation plan will be made. After the laboratory part of the course is implemented, based on the students feedback, future work will be done on program revision and improvements.

### 5.1. Testing Environment

An open-source FC is mandatory for a multirotor platform dedicated to education. There are several FC families that support open-source software such as Pixhawk, Matek, Kakute, Omnibus. They vary in size, weight, processor speed, amount of memory, number and type of I/O interfaces, supported features, open or closed hardware, supported peripherals and supported software tools. In this research, Pixhawk FC (ProfiCNC, Black Hill, Australia) was used as the open-source hardware and software control module (avionics) based on PX4 [45] ecosystem. Some of the key features of the PX4 ecosystem are modular software architecture, open-source platform, proven system, interoperability, and permissive license.

The used FCs were Pixhack as a derivative of Pixhawk 1 and Pixhawk Cube. Additional convenience of Pixhawk FCs is that they come with a “carrier board” that reduces wiring and contributes to the ease of use of ports, or connection to peripherals. Furthermore, the 14 PWM output ports give the possibility of connection to a large number of actuators. It also supports a variety of communication protocols (CAN, I2C, UART, SPI) to connect to various peripheral devices, such as remote control (RC), telemetry, or global positioning system (GPS) module. The controller communicates with telemetry and GPS via the UART protocol, while the RC receiver, which receives signals from the RC transmitter (operator joystick), exchanges data with the controller via the S.BUS protocol. The accelerometer, magnetometer, and gyroscope sensors, with triple redundancy, are integrated inside the controller and isolated from vibrations. The Cube has a powerful 32-bit 168 MHz STM32F427 Cortex M4 processor, 256 kB of RAM, and 2 MB of flash memory. To perform tasks that require more processing power (e.g., for computer vision data processing), there is the possibility of connecting the Cube to an additional controller. One such supported and the commonly used controller is Edison.

PX4 software is written in C and C++ languages, and as mentioned before, PX4 is modular, where adding new modules/applications can add new functionality to a particular system. In recent years, PX4 has also received support through MATLAB/Simulink in form of a toolbox that allows the interaction of Simulink and Pixhawk FCs. In other words, control algorithms can be developed, built, and uploaded to the Pixhawk FC, while other functionalities of the PX4 system remain intact. Such an approach offers intuitive development, simple interaction, and graphical representation of control algorithms, with basic programming skills. Real-time interaction with the UAV is also possible. Due to the mentioned benefits, such a system is a great testing environment for educational purposes. Students can focus on developing control algorithms and disseminating knowledge. In addition, interaction with the UAV in real-time speeds up the learning process. By changing the parameters/settings in the UAV model, the student can see the results of the change almost instantly.

**Figure 13:**
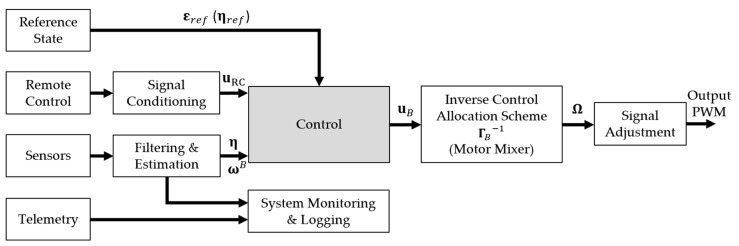
PX4 controller schematic.

The central part of the PX4 controller is the control system block, which represents a control algorithm that calculates and sends control signals to the propulsion module based on the reference state and sensor data. Figure 13 shows a proposed scheme of a control module for multirotor configurations. The inputs to the control system are a reference state that can be a planned flight path, RC signals, and sensor readings. For full-state estimation, peripheral sensors, such as GPS and RTK GPS modules, are typically used to determine position. The telemetry module is used for two-way communication between the aircraft and the base station. The system monitoring and logger block are used to store flight data consisting of aircraft status, battery and propulsion status, etc. Since the case of attitude control is further considered for experimental validation, the state of the system is obtained from the IMU sensors integrated into the Pixhawk Cube FC. The signal conditioning block is used to adjust the RC signal. It is possible to implement a gain tuning module using the available channels on the RC transmitter, which can be used to adjust and test the controller parameters in real-time. Block filtering and estimation is used for filtering raw signals from sensors and estimation of different quantities depending on the purpose of the experiment.

### 5.2. Experimental Validation

**Figure 14:**
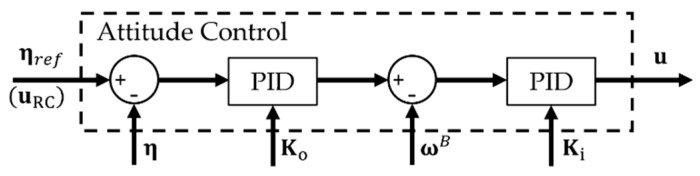
PX4 Control System Block.

The functionality of the proposed EMMR platform and the key elements of the first exercise planned to be implemented in the course on UAV mechatronics are presented and tested through the experiment. The first exercise covers the cascade PID controller shown in Figure 14. The goal of the exercise is to get acquainted with the real system, and the typical educational objective is to adjust the control parameters. For this purpose, the RC gain tuning module is implemented in the PX4 controller as part of the signal conditioning block. The module can tune the controller parameters in real-time based on the signal sent by the operator. Gain parameters of the controller for outer loop $K_{o}$, and inner loop $K_{i}$ were chosen empirically. The modular platform allows testing and analysis of different configurations on the same exercises. Before testing, it is necessary to assemble the considered configuration and implement the motor mixer.

**Figure 15:**
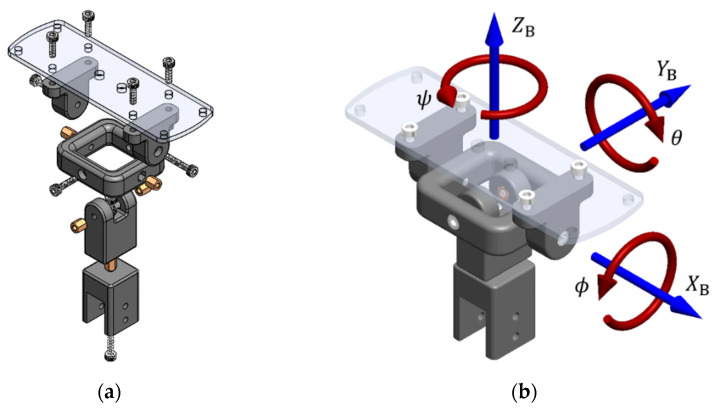
3 DOF gimbal joint: (**a**) parts; (**b**) assembly.

The derived prototype of the EMMR was experimentally tested on a setup with a three DOF gimbal joint (only change of orientation enabled). The gimbal joint consists of four main parts (Figure 15a) connected with screw connections that also serve as rotation axes (Figure 15b), which enables a simple and inexpensive assembly. The main goal of joint design is to keep the axes as close as possible to the multirotor main axes of inertia. The dimensions of the presented gimbal joint can easily be scaled up or down.

Experimental testing of attitude control was performed on several multirotor UAV configurations by setting various reference orientations during the testing cycle. In the first case, testing was performed for conventional flat quadrotor configuration (FX4) with 7024 propellers using a power source whose voltage was equivalent to a fully charged 3S battery. A motor mixer of quadrotor, which is implemented in PX4 controller, is given by the following expression:

(18) $$\left[\begin{array}{l} {\omega_{1}}^{2}\\ {\omega_{2}}^{2}\\ {\omega_{3}}^{2}\\ {\omega_{4}}^{2} \end{array}\right]=\left[\begin{array}{cccccc} 0 & 0 & \frac{1}{4k_{f}} & -\frac{\sqrt{2}}{4k_{f}l} & -\frac{\sqrt{2}}{4k_{f}l} & -\frac{1}{4k_{\tau }}\\ 0 & 0 & \frac{1}{4k_{f}} & \frac{\sqrt{2}}{4k_{f}l} & \frac{\sqrt{2}}{4k_{f}l} & -\frac{1}{4k_{\tau }}\\ 0 & 0 & \frac{1}{4k_{f}} & \frac{\sqrt{2}}{4k_{f}l} & -\frac{\sqrt{2}}{4k_{f}l} & \frac{1}{4k_{\tau }}\\ 0 & 0 & \frac{1}{4k_{f}} & -\frac{\sqrt{2}}{4k_{f}l} & \frac{\sqrt{2}}{4k_{f}l} & \frac{1}{4k_{\tau }} \end{array}\right]\left[\begin{array}{l} f_{x}\\ f_{y}\\ f_{z}\\ \tau_{\phi }\\ \tau_{\theta }\\ \tau_{\psi } \end{array}\right].$$

**Figure 16:**
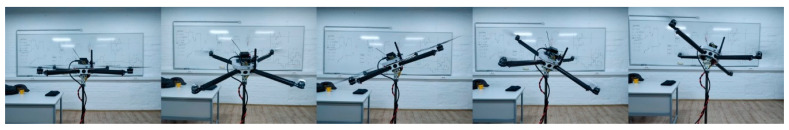
Experimental testing of attitude control on conventional flat quadrotor configuration (FX4).

Figure 16 shows five different reference orientations determined by the roll angle (ϕ) around $X_{B}$ axis, and pitch angle (θ) around $Y_{B}$ axis as shown in Figure 15b. The first reference orientation is defined by angles ϕ=0° and θ=0°, the second by angles ϕ=0° and θ=−20°, the third by ϕ=−20° and θ=0°, the fourth by ϕ=20° and θ=−20°, and the fifth by angles ϕ=20° and θ=20°.

**Figure 17:**
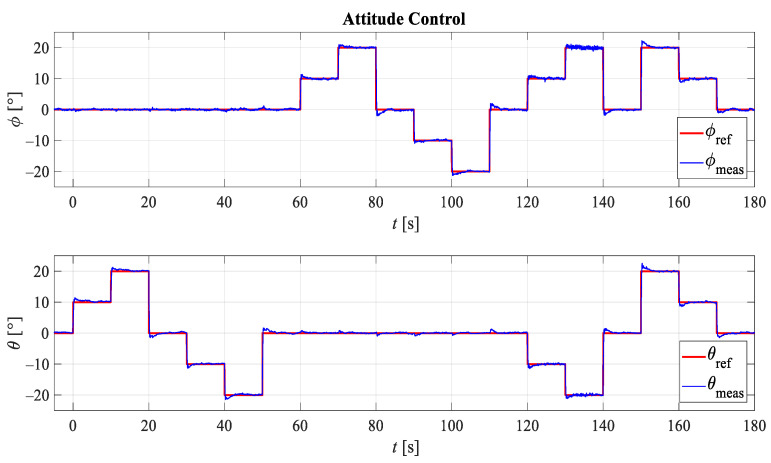
Experimental results of flat quadrotor configuration (FX4) response for attitude control.

**Figure 18:**
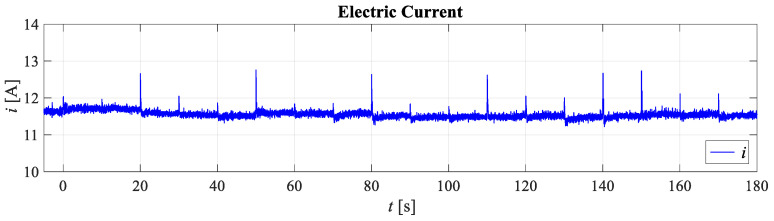
Experimental results of flat quadrotor configuration (FX4) energy consumption.

Figure 17 and Figure 18 show the results of the experimental testing for the first case. Since attitude control was considered, the system response with respect to the reference roll angle and the reference pitch angle is shown. Figure 18 shows the electricity consumption, more precisely the fluctuation of the electric current.

In the second case, testing was performed for conventional flat hexarotor configuration (FX6) with 7024 propellers using the same power source with voltage equivalent to a fully charged 3S battery. A motor mixer of hexarotor is given by the following expression.

**Figure 19:**
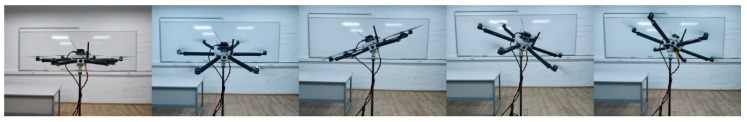
Experimental testing of attitude control on flat hexarotor configuration (FX6).

Figure 19 shows five different reference orientations which are the same as for the first case. From the presented EMMR platform it is possible to assemble and test other conventional configurations in addition to the ones previously shown.

(19) $$\left[\begin{array}{l} {\omega_{1}}^{2}\\ {\omega_{2}}^{2}\\ {\omega_{3}}^{2}\\ {\omega_{4}}^{2}\\ {\omega_{5}}^{2}\\ {\omega_{6}}^{2} \end{array}\right]=\left[\begin{array}{cccccc} 0 & 0 & \frac{1}{6k_{f}} & -\frac{1}{3k_{f}l} & 0 & \frac{1}{6k_{\tau }}\\ 0 & 0 & \frac{1}{6k_{f}} & \frac{1}{3k_{f}l} & 0 & -\frac{1}{6k_{\tau }}\\ 0 & 0 & \frac{1}{6k_{f}} & \frac{1}{6k_{f}l} & -\frac{\sqrt{3}}{6k_{f}l} & \frac{1}{6k_{\tau }}\\ 0 & 0 & \frac{1}{6k_{f}} & -\frac{1}{6k_{f}l} & \frac{\sqrt{3}}{6k_{f}l} & -\frac{1}{6k_{\tau }}\\ 0 & 0 & \frac{1}{6k_{f}} & -\frac{1}{6k_{f}l} & -\frac{\sqrt{3}}{6k_{f}l} & -\frac{1}{6k_{\tau }}\\ 0 & 0 & \frac{1}{6k_{f}} & \frac{1}{6k_{f}l} & \frac{\sqrt{3}}{6k_{f}l} & \frac{1}{6k_{\tau }} \end{array}\right]\left[\begin{array}{l} f_{x}\\ f_{y}\\ f_{z}\\ \tau_{\phi }\\ \tau_{\theta }\\ \tau_{\psi } \end{array}\right].$$

**Figure 20:**
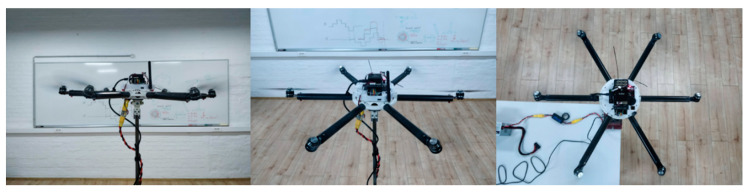
Experimental testing of attitude control on fully-actuated non-flat hexarotor configuration (NFX6).

Since the EMMR also allows the assembly of configurations with a non-flat (non-planar) geometric arrangement of rotors, fully-actuated multirotor configurations that represent omnidirectional aerial robots can also be assembled. For this purpose, the outer ring was used which allows passive adjustment of the rotor tilt angle, thus determining the allocation of aerodynamic forces and rotor torques to the aircraft control vector. Preliminary tests (Figure 20) were performed on a fully-actuated hexarotor configuration with passively tilted rotors. In this case motor mixer is given by the following expression

(20) $$\left[\begin{array}{l} {\omega_{1}}^{2}\\ {\omega_{2}}^{2}\\ {\omega_{3}}^{2}\\ \omega_{4}{}^{2}\\ \omega_{5}{}^{2}\\ \omega_{6}{}^{2} \end{array}\right]=\left[\begin{array}{cccccc} \frac{1}{3k_{f}s_{\gamma }} & 0 & \frac{1}{6k_{f}c_{\gamma }} & -\frac{1}{3\left(k_{f}lc_{\gamma }-k_{\tau }s_{\gamma }\right)} & 0 & \frac{1}{6\left(k_{f}ls_{\gamma }+k_{\tau }c_{\gamma }\right)}\\ \frac{1}{3k_{f}s_{\gamma }} & 0 & \frac{1}{6k_{f}c_{\gamma }} & \frac{1}{3\left(k_{f}lc_{\gamma }-k_{\tau }s_{\gamma }\right)} & 0 & -\frac{1}{6\left(k_{f}ls_{\gamma }+k_{\tau }c_{\gamma }\right)}\\ -\frac{1}{6k_{f}s_{\gamma }} & \frac{\sqrt{3}}{6k_{f}s_{\gamma }} & \frac{1}{6k_{f}c_{\gamma }} & \frac{1}{6\left(k_{f}lc_{\gamma }-k_{\tau }s_{\gamma }\right)} & -\frac{\sqrt{3}}{6\left(k_{f}lc_{\gamma }-k_{\tau }s_{\gamma }\right)} & \frac{1}{6\left(k_{f}ls_{\gamma }+k_{\tau }c_{\gamma }\right)}\\ -\frac{1}{6k_{f}s_{\gamma }} & \frac{\sqrt{3}}{6k_{f}s_{\gamma }} & \frac{1}{6k_{f}c_{\gamma }} & -\frac{1}{6\left(k_{f}lc_{\gamma }-k_{\tau }s_{\gamma }\right)} & \frac{\sqrt{3}}{6\left(k_{f}lc_{\gamma }-k_{\tau }s_{\gamma }\right)} & -\frac{1}{6\left(k_{f}ls_{\gamma }+k_{\tau }c_{\gamma }\right)}\\ -\frac{1}{6k_{f}s_{\gamma }} & -\frac{\sqrt{3}}{6k_{f}s_{\gamma }} & \frac{1}{6k_{f}c_{\gamma }} & -\frac{1}{6\left(k_{f}lc_{\gamma }-k_{\tau }s_{\gamma }\right)} & -\frac{\sqrt{3}}{6\left(k_{f}lc_{\gamma }-k_{\tau }s_{\gamma }\right)} & -\frac{1}{6\left(k_{f}ls_{\gamma }+k_{\tau }c_{\gamma }\right)}\\ -\frac{1}{6k_{f}s_{\gamma }} & -\frac{\sqrt{3}}{6k_{f}s_{\gamma }} & \frac{1}{6k_{f}c_{\gamma }} & \frac{1}{6\left(k_{f}lc_{\gamma }-k_{\tau }s_{\gamma }\right)} & \frac{\sqrt{3}}{6\left(k_{f}lc_{\gamma }-k_{\tau }s_{\gamma }\right)} & \frac{1}{6\left(k_{f}ls_{\gamma }+k_{\tau }c_{\gamma }\right)} \end{array}\right]\left[\begin{array}{l} f_{x}\\ f_{y}\\ f_{z}\\ \tau_{\phi }\\ \tau_{\theta }\\ \tau_{\psi } \end{array}\right].$$

The experimental tests presented in the paper show only a part of the EMMR potential and are given as proof of concept. An attitude control experiment with a multirotor platform mounted on a gimbal has some similarities and relationships to the well-known ball-on-plate system and can be used as a benchmark platform for various control methods.

## 6. Discussion

For the purpose of research and education, small or micro aircraft are mainly used with different EPU components. Unlike some commercial [17] and experimental [18,27] aircraft, the propulsion module of the proposed EMMR platform consists of EPUs based on BLDC motors which are mainly used for multirotor UAVs of various sizes. One of the successful goals was to design a versatile modular multirotor platform (priced under €800) that can be easily produced and implemented in the teaching of existing or new mechatronics courses.

Compared to existing experimental multirotors, such as [25,26], the proposed EMMR has several advantages, mainly related to its versatility, ease of prototyping, and low manufacturing and repair costs. Due to the modular design, multirotor configurations with several geometric arrangements are supported. An additional advantage is the simple expansion of the aircraft capability with the addition of new modules and components. For example, the addition of new sensors for obstacle avoidance, such as infrared, ultrasonic or laser sensor arrays, or use of different equipment, such as parachute, retractable landing gear, LEDs, gimbal camera stabilizer, various gear for interaction with the environment and other. Besides that, the EMMR platform is suitable to test on various types of test rigs, as it is shown in combination with a three-axis gimbal joint which can be easily expanded with a load cell. Apart from the aforementioned advantages, unlike the test setups [24,27], after assembling the energy module and landing gear, the proposed EMMR is ready to fly multirotor UAV. The proposed modular design approach is also compatible with a large power range of EPU components and scalable. The propulsion components can be chosen freely because the motor brackets can be easily modified if needed and the size of the multirotor can be easily altered by changing the length of the carbon tubes that serves as multirotor arms.

In Table 3, a comparison of commercial, experimental, and conceptual platforms used for research in the field of control system design and aerial robotics with the proposed EMMR platform is given.

The EMMR platform can be used as a common four-rotor (quadrotor) experimental aircraft [23,25] for testing different control methods of underactuated systems [27,28]. Configurations with more than four rotors can also be assembled, such as hexarotor [29] used for teaching fault-tolerant control. In addition to the conventional configurations [30], the proposed EMMR also allows assembling fully-actuated non-planar configurations with passively tilted rotors. The ability to assemble fully-actuated configurations enables students and researchers experimental testing of omnidirectional aerial robots in missions that require complex movements. The feasibility of a fully-actuated configuration with passively tilted rotors has been demonstrated through preliminary tests. Bearing in mind the aforementioned possibilities of the proposed platform, it can be concluded that EMMR combines the existing configurations used in research and education in one embodiment. Therefore, it covers more educational applications than the platforms used so far, as shown in Table 2. For example, compared to a fully-actuated hexarotor aircraft used in research [33,34], the proposed EMMR can be used in the same way, but it also offers a possibility to assemble a fully-actuated octorotor or any other conventional configuration.

Laboratory exercises in the UAV mechatronics course were viewed as an area of the potential application of the EMMR platform as an educational tool. The possibilities of the proposed platform extend the educational objectives experimentally covered in the field of omnidirectional aerial robotics and the field of fault-tolerant control in terms of the diversity of the propulsion module. With the aim of additional validation of the considered course educational objectives, among complementary filter used in research [26], and built-in filters and estimators, new approach to state and disturbance estimation is tested and evaluated in [45] where the considered control system consists of algebraic ESO used for the monitoring of the system state and external disturbances. Such an estimator can be considered as a fundamental element of a robust control algorithm.

## 7. Conclusions

In this paper, a modular design approach is proposed for the development of a multirotor UAV engineering education platform. A comprehensive mathematical model of a multirotor UAV is presented, where the emphasis is on the control allocation scheme which generally describes multirotor configuration. The inverse of the control allocation scheme (motor mixer) allows control implementation for configurations with various geometric arrangements and propulsion parameters. The proposed modular concept consists of four separated subsystems that make up a multirotor UAV system. In addition to the fact that the proposed design allows easy replacement of the platform subsystems and enables the development of an easy-to-maintain and repair engineering education platform, the main feature is the versatility of a propulsion subsystem. The process of EMMR development takes place through the phases of computer-aided design and rapid prototyping, which allows easy and fast reproduction of the platform parts.

For educational and research applications, an open-source control module is a must, while open-architecture is also preferable. In this research, the control module based on Pixhawk FC has been used which has an additional feature of compatibility with the MATLAB/Simulink software package commonly used in the academic community for mechatronics courses. In order to validate the proposed EMMR platform, experimental testing of different aircraft configurations was performed and the results for the aircraft attitude control were presented. It is planned to use the EMMR as an educational tool for performing exercises from the new course on UAV mechatronics at the University of Zagreb. Extensive experimental tests are planned in future research to validate the considered educational objectives based on which it is further planned to create a syllabus for course laboratory exercises.

## Figures and Tables

**Table 1:** Considered LiPo batteries [42].

Battery Parameters	Turnigy Nano-Tech Series—3S				
Capacity (mAh)	1800	2200	3000	4000	5000
Discharge rate (C)	30	25	30	25	25
Mass (g)	142	168	215	333	403

**Table 2:** Educational objectives in course related to UAV mechatronics.

Control Objectives	Controlled DOF		
	1–3	4	6
Stabilized remote control	Test bench	Underactuated configurations	Fully-actuated configurations
Trajectory tracking control			
Disturbance rejection control			
Fault-tolerant control			

**Table 3:** Comparison of commercial, experimental, and conceptual platforms.

Platform	Conventional Underactuated Multirotor Configurations			Non-Planar Fully-Actuated Multirotor Configurations	
	*N* = 4	*N* = 6	*N* = 8	*N* = 6	*N* = 8
IRIS quadrotor from 3DR [16]	YES	NO	NO	NO	NO
Quadrotor platform with foldable arms [26]	YES	NO	NO	NO	NO
Hexarotor platform [29]	NO	YES	NO	NO	NO
Multipurpose modular drone [30]	YES	YES	YES	NO	NO
Tilt-Hex platform [33]	NO	YES	NO	YES	NO
EMMR platform	YES	YES	YES	YES	YES

## Data Availability

Not applicable.
